# Inference of Ancestries and Heterozygosity Proportion and Genotype Imputation in West African Cattle Populations

**DOI:** 10.3389/fgene.2021.584355

**Published:** 2021-03-23

**Authors:** Netsanet Z. Gebrehiwot, Hassan Aliloo, Eva M. Strucken, Karen Marshall, Mohammad Al Kalaldeh, Ayao Missohou, John P. Gibson

**Affiliations:** ^1^Centre for Genetic Analysis and Applications, School of Environmental and Rural Science, University of New England, Armidale, NSW, Australia; ^2^International Livestock Research Institute and Centre for Tropical Livestock Genetics and Health, Nairobi, Kenya; ^3^L‘École Inter-États des Sciences et Médecine Vétérinaires de Dakar (EISMV), Dakar, Senegal

**Keywords:** ADMIXTURE, African cattle, global ancestry, Minimac, LAMP-LD, local ancestry, PCA, SNPs

## Abstract

Several studies have evaluated computational methods that infer the haplotypes from population genotype data in European cattle populations. However, little is known about how well they perform in African indigenous and crossbred populations. This study investigates: (1) global and local ancestry inference; (2) heterozygosity proportion estimation; and (3) genotype imputation in West African indigenous and crossbred cattle populations. Principal component analysis (PCA), ADMIXTURE, and LAMP-LD were used to analyse a medium-density single nucleotide polymorphism (SNP) dataset from Senegalese crossbred cattle. Reference SNP data of East and West African indigenous and crossbred cattle populations were used to investigate the accuracy of imputation from low to medium-density and from medium to high-density SNP datasets using Minimac v3. The first two principal components differentiated *Bos indicus* from European *Bos taurus* and African *Bos taurus* from other breeds. Irrespective of assuming two or three ancestral breeds for the Senegalese crossbreds, breed proportion estimates from ADMIXTURE and LAMP-LD showed a high correlation (*r* ≥ 0.981). The observed ancestral origin heterozygosity proportion in putative F1 crosses was close to the expected value of 1.0, and clearly differentiated F1 from all other crosses. The imputation accuracies (estimated as correlation) between imputed and the real data in crossbred animals ranged from 0.142 to 0.717 when imputing from low to medium-density, and from 0.478 to 0.899 for imputation from medium to high-density. The imputation accuracy was generally higher when the reference data came from the same geographical region as the target population, and when crossbred reference data was used to impute crossbred genotypes. The lowest imputation accuracies were observed for indigenous breed genotypes. This study shows that ancestral origin heterozygosity can be estimated with high accuracy and will be far superior to the use of observed individual heterozygosity for estimating heterosis in African crossbred populations. It was not possible to achieve high imputation accuracy in West African crossbred or indigenous populations based on reference data sets from East Africa, and population-specific genotyping with high-density SNP assays is required to improve imputation.

## Introduction

Indigenous cattle in Africa are an important genetic resource for diverse human communities, providing products and by-products, such as food, wealth, and economic security (Okomo-Adhiambo, 2002). Genetic improvement programs using artificial selection within the local population are one method to improve productivity (Effa et al., 2009; Tegegne et al., 2010). Crossbreeding of locally adapted cattle with high-yielding European dairy breeds is an alternative strategy to improve productivity and improve the livelihoods of African smallholder farmers in a relatively short period (Wuletaw, 2004; Tegegne et al., 2010). Crossbreeding can increase dairy cattle production by creating new combinations of genotypes of different breeds to optimize the additive and heterotic genetic expression and achieve the desired balance of productivity and adaptation trait expression (Gregory and Cundiff, 1980; Simm, 1998).

The level of extra heterosis in crossbreds compared to purebreds is a function of the degree of heterozygosity for the origin of alleles from the ancestral populations, referred to as ancestral origin heterozygosity in this study. In a homogeneous crossbred population that results entirely from *inter-se* crossing, the level of ancestral origin heterozygosity is a function of the breed composition. In other crossbred populations, the level of ancestral origin heterozygosity depends on the breed composition of the parents of an individual (McAllister, 2002). For example, an F1 cross has an ancestral origin heterozygosity of 1.0, which is twice the ancestral origin heterozygosity and hence twice the expected heterosis of an F2 cross, even though they have identical breed composition. In order to estimate the level of heterosis in crossbred populations, one needs to have an estimate of the ancestral origin heterozygosity for each individual that is recorded and available for genomic evaluation. An estimate of breed composition and ancestral origin heterozygosity can be obtained from complete pedigree information, but pedigree information is unavailable in most smallholder crossbred dairy populations (Rege, 2001). An alternative is to genotype animals for large numbers of SNPs and use this information to estimate breed composition and heterozygosity.

Molecular genetic markers, most recently SNPs, can be used to estimate the genetic ancestry of individuals. Methods embedded in software such as ADMIXTURE (Alexander et al., 2009) or STRUCTURE (Pritchard et al., 2000; Falush et al., 2003) estimate global ancestry, i.e., the ancestral breed proportions averaged across the whole genome. These software programs do not provide estimates of ancestral origin heterozygosity. Methods such as Lanc-CSV (Brown and Pasaniuc, 2014), LAMP-LD (Pasaniuc et al., 2009; Baran et al., 2012), and MULTIMIX (Churchhouse and Marchini, 2013) provide estimates of local ancestry, i.e., the breed origin of haplotypes, and hence breed proportion at every point in the genome. This allows ancestral origin heterozygosity to be estimated at every point in the genome and hence also the average ancestral origin heterozygosity of an individual.

Local ancestry mapping, using the LAMP software (Sankararaman et al., 2008), was employed in African cattle populations by Flori et al. (2014) and Bahbahani et al. (2015) to examine whether their SNP-based signatures of selection showed a bias to either of the two assumed ancestral populations. The LAMP software was also used by Khayatzadeh et al. (2018) to assign ancestral origin of SNP genotypes in a European admixed cattle population, allowing SNP dominance effects and epistatic loss to be estimated. The African populations we study here evolved from one or two (African *Bos taurus* and African zebu, respectively) or three (crossbred dairy populations) principal ancestral populations. We used LAMP-LD, which performs better than LAMP when there are more than two ancestral populations (Baran et al., 2012) to estimate global and local ancestry in these populations.

Crossbreeding and selection are important synergic approaches to improve production in the long-term. In the absence of pedigree recording in most indigenous and crossbred dairy populations, high-density SNP genotypes can be used to generate a genomic relationship matrix (GRM), enabling genetic improvement to be rapidly implemented (VanRaden, 2008). However, genomic selection requires the routine genotyping of a large number of recorded individuals and selection candidates, which can be expensive. A strategy to increase genotypic information while reducing testing costs is to genotype a large number of individuals with a lower-density assay and impute to higher density genotypes (Khatkar et al., 2012; Wiggans et al., 2012; Berry et al., 2014).

Several software programs have been developed for SNP imputation. These are mainly based either on linkage disequilibrium (LD) information such as Beagle (Browning and Browning, 2007), IMPUTE2 (Howie et al., 2009), MaCH (Li et al., 2010), Minimac (Howie et al., 2012); or on a combination of LD and family or pedigree information such as Dagphase (Druet and Georges, 2010), FImpute (Sargolzaei et al., 2011), AlphaImpute (Hickey et al., 2012), and FindHap (VanRaden et al., 2011).

Recently, Aliloo et al. (2018) assessed the genotype imputation accuracy in 3,083 East African crossbred cattle genotyped with the Illumina 777k SNP assay, using FImpute v2.2 (Sargolzaei et al., 2014), Beagle v4.1 (Browning and Browning, 2016), and Minimac v3 (Das et al., 2016) and found that Minimac v3 and a reference set that combines crossbred and ancestral purebred animals generally gave the highest accuracy of imputations. But this study provided no information about whether data from East African crossbreds would be useful in the imputation of other crossbred populations in Africa or for indigenous populations. The accuracy of genotype imputation across populations is highly affected by the LD and persistence of the LD phase between populations, which has not been assessed for African indigenous or crossbred populations. Berry and Kearney (2011) have documented that the degree of relationship between validation and reference populations is one of the factors affecting imputation accuracy. Therefore, it is necessary to estimate the ancestral background of the indigenous and crossbred populations to make an informed decision about which animals and breeds to best use as reference populations.

The overall objective of the current study was to assess the ability to infer genotypes and genotype ancestry in African populations based on diverse and local information as enablers of a range of genetic improvement applications. The study investigates: (1) Inference of global and local ancestry in West African crossbreds to obtain substantially more information on their genetic history. ADMIXTURE and PC analyses were performed to estimate the global ancestry, while LAMP-LD was used for local ancestry inference with different approaches in West African crossbreds. We then compared the performance of global and local ancestry inference methods; (2) Estimation of ancestral origin and individual heterozygosity proportions in West African crossbreds. The ancestral origin heterozygosity proportion was calculated from the local ancestry inferences obtained from LAMP-LD, while the individual heterozygosity was calculated across all loci which are heterozygous; (3) Accuracy of genotype imputation in African indigenous and West African crossbred cattle populations when imputing from low and medium-density to high-density SNP panels, using East and West African reference populations separately or combined. This is the first imputation study considering African indigenous and West African crossbred populations.

## Materials and Methods

### Animals

SNP genotype data of 4,291 animals representing European *Bos taurus* dairy breeds, East and West African indigenous and crossbred dairy cattle sampled from different countries were used for this study (Table 1). These data were obtained from several public-domain databases, plus projects run by the International Livestock Research Institute (ILRI) and collaborators (Marshall et al., 2017, 2020; Ema et al., 2018), and the Genomics Reference Resource for African Cattle (GRRFAC) supported by the Centre for Tropical Livestock Genetics and Health (CTLGH), and the Dairy Genetics East Africa project (DGEA; Strucken et al. (2017). The breed classifications of the West African crossbred animals were based on farmers’ and enumerators’ assumptions as well as, where available, recorded sire and dam information. These crossbred animals were classified as undefined crossbreds or as crosses between the local breed Gobra with Holstein-Friesian, Montbéliarde, or Normande.

**TABLE 1:** Animal populations, numbers, and sources.

Breed	Population group	Origin/country	Number of animals	Array (Illumina)	Genotype source
Friesian	EuB.t	United Kingdom	25	BovineHD	SRUC
Guernsey	EuB.t	United States and United Kingdom	20	BovineHD	Bovine HapMap Consortium et al., 2009
Holstein	EuB.t	United States and NZ	20	BovineHD	Bovine HapMap Consortium et al., 2009
Jersey	EuB.t	United States and NZ	20	BovineHD	Bovine HapMap Consortium et al., 2009
Montbéliarde	EuB.t	France	20	BovineSNP50	Decker et al., 2014
Pooled *Bos indicus*	B.i	India	105	BovineHD	Strucken et al., 2019
N’Dama	WAI	Guinea	20	BovineHD	Bovine HapMap Consortium et al., 2009
N’Dama	WAI	Senegal	14	BovineHD	GRRFAC
N’Dama1	WAI	Cote d’Ivoire	20	BovineSNP50	Decker et al., 2014
N’Dama2	WAI	Southeast Burkina Faso	14	BovineSNP50	Decker et al., 2014
N’Dama3	WAI	Southwest Burkina Faso	17	BovineSNP50	Decker et al., 2014
Lagune	WAI	Benin	20	BovineSNP50	Decker et al., 2014
Lagunaire	WAI	West Africa	5	BovineHD	Bovine HapMap Consortium et al., 2009
Somba	WAI	Togo	20	BovineSNP50	Decker et al., 2014
Baoule	WAI	Burkina Faso	20	BovineSNP50	Decker et al., 2014
Baoule	WAI	Burkina Faso	19	BovineHD	GRRFAC
Djakore*	WAI	Senegal	7	BovineSNP50	Marshall et al., 2020
Gobra*	WAI	Senegal	118	BovineSNP50	Marshall et al., 2020
Gobra*	WAI	Senegal	14	BovineHD	GRRFAC
Maure*	WAI	Senegal	12	BovineSNP50	Marshall et al., 2020
Maure*	WAI	Senegal	15	BovineHD	GRRFAC
Gobara × Maure*	WAI	Senegal	10	BovineSNP50	Marshall et al., 2020
Gobara × Guzerat*	WAI	Senegal	31	BovineSNP50	Marshall et al., 2020
Bororo	WAI	Chad	20	BovineSNP50	Decker et al., 2014
Fulani	WAI	Benin	20	BovineSNP50	Decker et al., 2014
Kuri	WAI	Chad	20	BovineSNP50	Decker et al., 2014
Borgou	WAI	Benin	20	BovineSNP50	Decker et al., 2014
Undefined indigenous	WAI	Senegal	66	BovineSNP50	Marshall et al., 2020
Ankole	EAI	Uganda	35	BovineHD	DGEA
SEAZ	EAI	Kenya	21	BovineHD	DGEA
Boran	EAI	Kenya	28	BovineHD	DGEA
Danakil-Harar	EAI	Ethiopia	30	BovineHD	DGEA
Begait-Barka	EAI	Ethiopia	30	BovineHD	DGEA
Boran	EAI	Ethiopia	28	BovineHD	DGEA
Iringa-Red	EAI	Tanzania	13	BovineHD	DGEA
Singida-White	EAI	Tanzania	22	BovineHD	DGEA
Sheko	EAI	Ethiopia	18	BovineHD	Bovine HapMap Consortium et al., 2009
Kenyan crossbred	EXX	Kenya	1,378	BovineHD	DGEA
Uganda crossbred	EXX	Uganda	555	BovineHD	DGEA
Ethiopia crossbred	EXX	Ethiopia	545	BovineHD	DGEA
Tanzania crossbred	EXX	Tanzania	462	BovineHD	DGEA
Senegal crossbreed	WXX	Senegal	253	BovineSNP50	Marshall et al., 2020
Senegal crossbreed	WXX	Senegal	141	BovineHD	CTLGH
Total			4,291		

*Senegalese indigenous populations used in the pooled indigenous population (*N* = 105), EuB.t = European *Bos taurus*, B.i = *Bos indicus*, WAI = West African indigenous, EAI = East African indigenous, WXX = West African crossbreds, EXX = East African crossbreds, USA = United States of America, UK = United Kingdom, NZ = New Zealand, SRUC = Scottish Rural University College, CDN = Canadian Dairy Network.

### Genotyping and Quality Control

The samples were genotyped on either the Illumina BovineSNP50 BeadChip array (Illumina Inc., San Diego, CA, USA) comprising 54,609 SNPs or the Illumina BovineHD Beadchip (Illumina Inc., San Diego, CA, USA) containing 777,962 SNPs, as presented in Table 1. Data from the Bovine HapMap Consortium et al. (2009) and the 50k data from Decker et al. (2014) were obtained post quality control. Genotypes from the DGEA project and Scotland’s Rural College (SRUC) data were filtered using “SNPQC” an R pipeline (Gondro et al., 2014), retaining SNPs that had a median GC score >0.6 and a call rate >90%. The data from Senegal smallholder farms (Marshall et al., 2017, 2020; Ema et al., 2018) were processed for quality control using the GenABEL package (Aulchenko et al., 2007) in R Core Team (2018), retaining SNPs and animals with call rates >90%. Data from CTLGH and GRRFAC were quality controlled, including a median GC score >0.6 and a call rate >0.90%. In all datasets, only autosomal SNPs were included in this study.

The datasets were merged, keeping only common SNPs (37,632 SNPs) between the reference (detailed below) and West African crossbred populations for inference of global and local ancestry and estimates of heterozygosity proportions (dataset 1). For the genotype imputation, SNPs that had a minor allele frequency (MAF) lower than 0.01 were removed from medium and high-density datasets. FImpute V 2.2 (Sargolzaei et al., 2014) was used to impute the sporadically missing genotypes of individuals to have complete datasets for all animals at all loci. The number of SNPs retained was 28,649 from medium-density (dataset 2), and 621,309 SNPs from high-density panels (dataset 3) across 29 *B. taurus* autosomes based on UMD 3.1 genome assembley (Zimin et al., 2009).

### Global Ancestry Inference of West African Crossbred Animals

The global ancestry inference is important to estimate the fraction of ancestry contributed by each ancestral population as averaged across the entire genome. In this study, the global ancestry inference was undertaken using Senegalese (West African) crossbred populations. The reference populations were African *B. taurus* breeds (N’Dama, N’Dama1, Lagune, Baoule, and Lagunaire, *N* = 87), European *B. taurus* dairy breeds (Guernsey, Holstein, Jersey, Friesian, and Montbéliarde, *N* = 105), and a pooled *Bos indicus* population (*N* = 105). The pooled *Bos indicus* sample included 12 *Bos indicus* breeds from India, selected from 525 indigenous samples such that within breed relationships were minimal (Aliloo et al., 2020). The pooled indigenous reference population was from Senegal (Gobra, Maure, Djakore, hybrid animals between Gobra and Maure, and Gobra and Guzerat, *N* = 105), and the number of indigenous animals were reduced to make similar population size with other reference groups (also used in heterozygosity estimation). The African *B. taurus*, *Bos indicus*, and indigenous reference animals are those with zero European *B. taurus* breed proportion as determined by prior ADMIXTURE and PC analyses (Gebrehiwot, 2020; Gebrehiwot et al., 2020).

A maximum likelihood model, as implemented in the software ADMIXTURE 1.23 (Alexander et al., 2009), was used to estimate the global ancestry proportions of crossbred animals. ADMIXTURE was used in two alternatives supervised analyses where the ancestral reference populations were a pooled sample of European *B. taurus* and a pooled sample of indigenous breeds from Senegal (two-way admixture) (1), and African *B. taurus* populations, *Bos indicus*, and European *B. taurus* dairy breeds (three-way admixture) (2). These reference populations were chosen based on the ancestral information of Senegalese crossbreds as detailed by Gebrehiwot et al. (2020) and Gebrehiwot (2020).

The principal component analysis (PCA) was performed to explore and visualize the genetic variation between West African indigenous and crossbred animals and the reference populations. The PCA was based on a GRM constructed from SNP data according to the first method of VanRaden (2008) and calculated as:

$$\text{GRM}=Z\,Z^{\prime }/d$$

where the scaling parameter d was:

$$d=2*\sum \left(p_{i}*\left(1-p_{\text{i}}\right)\right)$$

The centered genotype matrix (**Z**) was constructed by subtracting the **P** matrix from the genotype matrix **M**, where *P* = 2*(*p*_*i*_−0.5), and *p*_*i*_ is the allele frequency at locus *i*.

### Local Ancestry Estimation in West African Crossbred Animals

The genome of admixed individuals resembles a mosaic of chromosomal regions originating from different ancestral populations. Finding the regional ancestry at each genomic location provides more information than the usual estimation of global ancestry alone (Padhukasahasram, 2014). Here, LAMP-LD software (Pasaniuc et al., 2009; Baran et al., 2012) was used to estimate the locus-specific ancestry of West African crossbreds in two scenarios of ancestry mapping. The two scenarios were two-way and three-way admixtures, using the same ancestral populations as for the global ancestry inference (see above). To infer the local ancestry, the dataset was first phased using Eagle v2.3.5 (Loh et al., 2016). The local ancestries of admixed animals were obtained from LAMP-LD with a window size of 12 SNPs and 15 as the number of states. LAMP-LD infers the ancestry in each window based on a likelihood model to trace the origins of admixed populations based on the haplotype patterns in ancestral reference populations.

### Estimation of Heterozygosity Proportion

Estimation of heterozygosity proportion in West African crossbred populations was undertaken using two approaches. Individual heterozygosity was calculated across all loci, scored as “1” if an individual was heterozygous at a locus and “0” for each homozygous locus; the mean across all loci was then recorded. The ancestral origin heterozygosity proportion was calculated from the local ancestry inferences obtained from LAMP-LD. Each haplotype of a given crossbred individual was scored as “1” if it was a heterozygous state of European *B. taurus* and indigenous ancestry (two-way), or African *B. taurus* or *Bos indicus* versus European *B. taurus* ancestry (three-way), and scored “0” otherwise. The sum of these scores was divided by the number of loci to obtain the average ancestral origin heterozygosity across the genome.

### Upper and Lower Limits of Heterozygosity

In crosses between two populations, the ancestral origin heterozygosity has upper and lower bounds that depend on the breed proportions of the crossbred animal and the breed proportions of its parents. The expectations can be obtained as the expected frequency of heterozygotes at a single locus, if the two ancestral parents are fixed for opposite alleles at that locus. For example, the ancestral origin heterozygosity of the two parental populations is zero. That for an F1 is exactly 1, which is the upper bound of heterozygosity, while that of an F2, resulting from the mating of two F1 animals is expected to be 0.5, which is the lower bound of heterozygosity for animals with 50% ancestry from each parent. The upper bound of the expected heterozygosity applies to all crossbreds that have at least one parent being a purebred ancestor. The lower bound applies to all *inter-se* matings between crossbred parents that have identical ancestral breed composition. The expected ancestral heterozygosity of all other crosses between crossbred parents lies between the upper and lower bounds of ancestral origin heterozygosity for animals of that breed composition.

Analogous bounds can be obtained for individual heterozygosity when it is assumed that all the animals of a given ancestral pure breed have the same heterozygosity. In this case expected heterozygosity can be considered as a trait whose expectation is the sum of additive genetic and heterosis effects. If *H*_1_ and *H*_2_ are the heterozygosity of parent breeds 1 and 2, respectively, and *p*_*i*_ is the breed proportion of parent breed 2 and *a*_*i*_ is the expected ancestral origin heterozygosity of crossbred animal *i*, then the expected heterozygosity of that animal, *H*_*i*_, is:

H=iH+1p(H-2H)1i+axi

where *x* = *H*_*F1*_ – *H*_1_ if *p* < 0.5 and *x* = *H*_*F1*_ – *H*_2_ if *p* > 0.5, and *H*_*F1*_ is the average individual heterozygosity of F1 animals. The upper and lower bounds for ancestral and individual heterozygosity are used in the results to illustrate the utility of ancestral versus individual heterozygosity as a useful metric in the estimation of genetic parameters of performance in crossbred populations.

### Genotype Imputation in West African Cattle Populations

Imputation was undertaken using a population-based algorithm, Minimac v3 (Das et al., 2016) with pre-phased data from Eagle v2.3.5 (Loh et al., 2016). Minimac v3 was chosen for genotype imputation because it provided the highest imputation accuracy in East Africa crossbred populations compared to FImpute and Beagle (Aliloo et al., 2018).

#### SNP Information for Imputation

The SNPs in common between the medium-density genotypes (dataset 2) and the commercially available Illumina BovineLD v2 SNP array (containing 7,931 SNPs) were retained to create the low-density dataset. There were 5,043, 28,649, and 621,309 SNPs in low-density, medium-density (dataset 2) and high-density (dataset 3) datasets, respectively.

#### Genotype Imputation Scenarios

Imputation was undertaken within and across geographical regions focussing mainly on West Africa using East African populations as a reference. As detailed in Table 2, a total of 36 imputation scenarios were considered to impute West African indigenous and crossbred populations, while four scenarios were used to impute East African indigenous and crossbred populations. Half of the imputation scenarios (18) were designed to investigate the imputation accuracies from low-density to medium-density SNP panels, and the other half was used for the imputation from medium-density to high-density SNP panels (Table 2). Based on the geographical regions where the reference populations were sampled from, the 36 imputation scenarios could be classified into three major groups based on the reference sets: using East African indigenous and crossbred individuals combined or separately (Scenario 1), using West African indigenous and crossbred individuals combined or separately (Scenario 2), and using a combination of East and West African indigenous and crossbred individuals (Scenario 3).

**TABLE 2:** Scenarios and the number of animals used in the reference and validation sets to assess imputation accuracy.

Scenario	Population in reference	Population in validation	LD-MD		MD-HD	
				
			Number in reference	Number in validation	Number in reference	Number in validation
Scenario 1						
1A	EAI	WAI	228	485	228	87
1B	EXX	WXX	2,982	394	2,982	141
1C	EAI + EXX	WAI	228 + 2,982	485	228 + 2,982	87
1D	EAI + EXX	WXX	229 + 2,982	394	229 + 2,982	141
1E	EAI	EAI	182	46	182	46
1F	EXX	EXX	2,385	597	2,385	597
Scenario 2						
2A	WAI	WAI	388	97	69	18
2B	WAI	WXX	388	79	69	29
2C	WXX	WAI	315	97	112	18
2D	WXX	WXX	315	79	112	29
2E	WAI + WXX	WAI	388 + 315	97	69 + 112	18
2F	WAI + WXX	WXX	388 + 315	79	69 + 112	29
Scenario 3						
3A	WAI + EAI	WAI	388 + 228	97	69 + 228	18
3B	WAI + EAI	WXX	388 + 228	79	69 + 228	29
3C	WXX + EXX	WAI	315 + 2,982	97	112 + 2,982	18
3D	WXX + EXX	WXX	315 + 2,982	79	112 + 2,982	29
3E	WAI + EAI + WXX + EXX	WAI	388 + 228 + 315 + 2,982	97	69 + 228 + 112 + 2,982	18
3F	WAI + EAI + WXX + EXX	WXX	388 + 228 + 315 + 2,982	79	69 + 228 + 112 + 2,982	29

*WAI = West African indigenous, EAI = East African indigenous, WXX = West African crossbreds, EXX = East African crossbred.*

To assess the imputation accuracy, direct imputation was performed for Scenario 1 and five-fold cross-validation for Scenarios 2 and 3. The target individuals for Scenarios 2 and 3 were randomly divided into five groups, and each group was used as a validation set, while the four remaining groups were used as a reference population.

Imputation accuracy was determined with two different criteria: (1) the allelic correlation of imputed versus real genotypes, and (2) the concordance rate computed as the ratio between the number of correctly imputed alleles versus the total number of imputed alleles.

## Results and Discussion

### Global and Local Ancestry Inferences in West African Crossbreds

**FIGURE 1:**
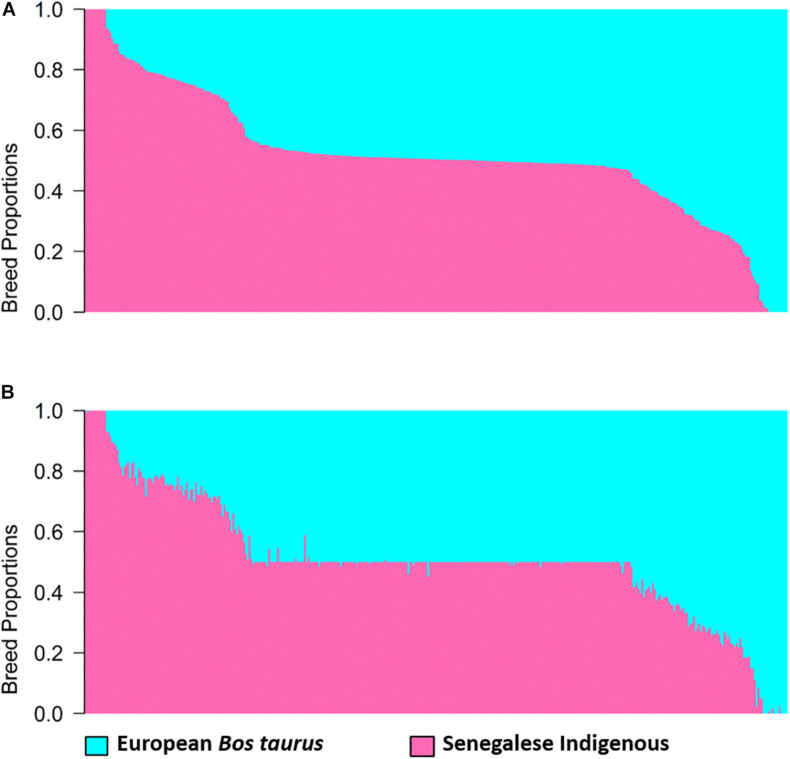
Estimates of breed proportion of West African crossbreds using two-way admixture from **(A)** ADMIXTURE and **(B)** LAMP-LD.

Estimates of global and local ancestry for the two-way admixture generated by ADMIXTURE and LAMP-LD, are shown in Figure 1. Each vertical bar represents an individual with the proportion of each ancestry depicted in a different color. The average European *B. taurus* and indigenous breed proportions estimated from ADMIXTURE (Figure 1A) were 0.481 (SD = 0.201) and 0.519 (SD = 0.201), respectively, and from LAMP-LD (Figure 1B) 0.491 (SD = 0.199), and 0.509 (SD = 0.199), respectively. The correlation between the breed proportion estimates obtained from the two algorithms was 0.995, showing that they have a strong association.

**FIGURE 2:**
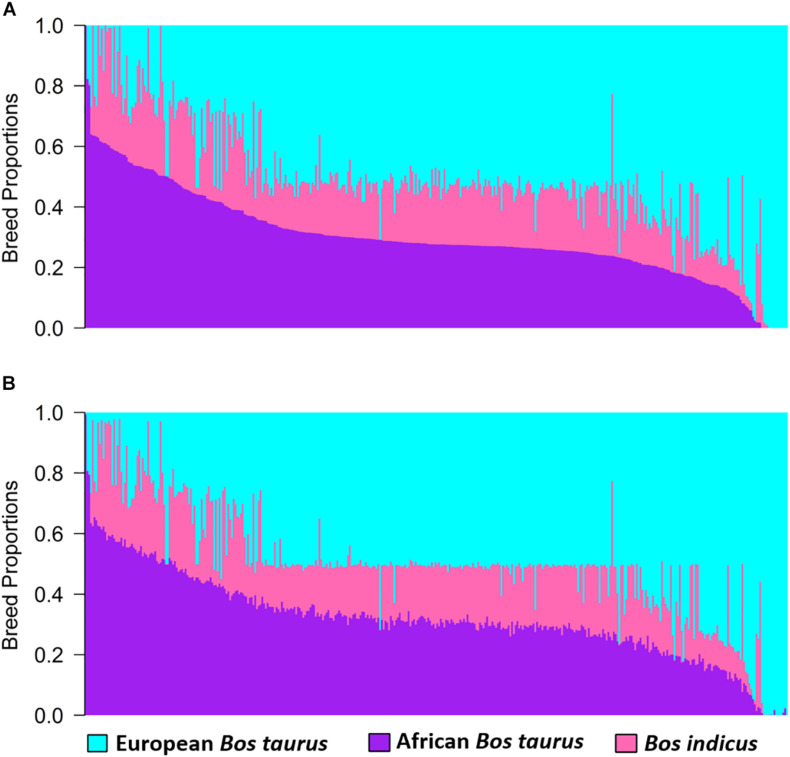
Estimates of breed proportion of West African crossbreds using three-way admixture from **(A)** ADMIXTURE and **(B)** LAMP-LD.

Estimates of global and local ancestry from the three-way admixture using ADMIXTURE and LAMP-LD are shown in Figure 2. The average European *B. taurus*, African *B. taurus*, and *Bos indicus* breed proportions from ADMIXTURE (Figure 2A) were 0.515 (SD = 0.199), 0.185 (SD = 0.091), and 0.300 (SD = 0.146), respectively. The average estimates of ancestral breed proportions from LAMP-LD (Figure 2B) were 0.501 (SD = 0.194), 0.181 (SD = 0.088) and 0.319 (SD = 0.143), respectively. The correlation between the estimates of the three breed proportions obtained from ADMIXTURE versus LAMP-LD were 0.994, 0.981, and 0.994, respectively. This correlation was consistent with previous results by Chen et al. (2014), who found that the LAMP-LD estimates showed a correlation of 0.989 with a supervised ADMIXTURE analysis in human populations. The estimates of average European breed proportion from ADMIXTURE and LAMP-LD for the three-way scenario were slightly higher (3.4 and 1%, respectively) than results for two-way admixture. Gebrehiwot et al. (2020) and Gebrehiwot (2020) found an average exotic dairy proportion of 0.503 (SD = 0.187) using twelve ancestral reference populations in a supervised ADMIXTURE analysis of West African crossbreds with overlapping data, which is consistent with the estimates here.

**FIGURE 3:**
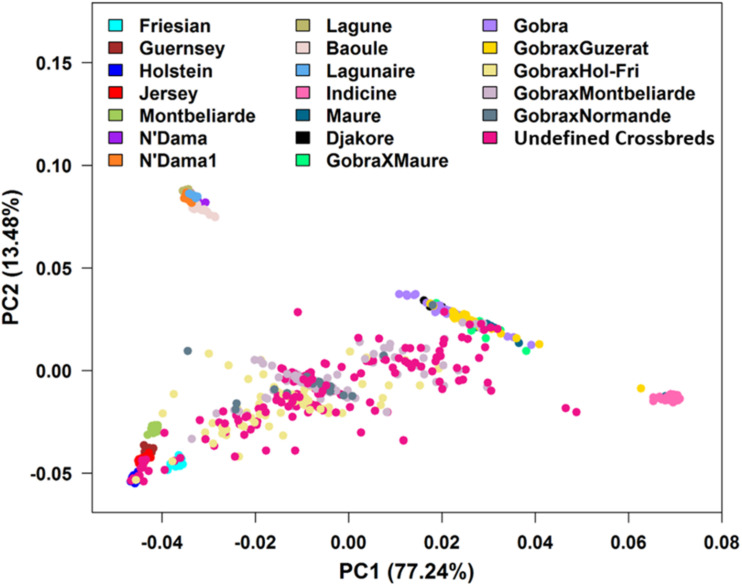
Plots of PC1 vs. PC2 for *Bos indicus*, African and European *Bos taurus*, West African indigenous and crossbred populations.

The PCA found that the first two PCs accounted for 77.24 and 13.48% of the total genetic variation in the GRM, differentiating *Bos indicus* from *B. taurus* and African *B. taurus* from other groups (Figure 3). This is consistent with the patterns found by several studies (Hanotte et al., 2002; Gautier et al., 2009; Kim et al., 2017; Verdugo et al., 2019; Gebrehiwot et al., 2020), analyzing various combinations of African indigenous and crossbred data along with the three reference groups. The *Bos indicus* reference populations clustered tightly together, showing that they are a pure *Bos indicus* population, while the African *B. taurus* populations clustered together with a few Baoule individuals appearing to be admixed with *Bos indicus*. The crosses between European dairy breeds and African indigenous breeds were distributed between the European and indigenous populations. A substantial number of Gobra × Holstein-Friesian, Gobra × Montbéliarde, Gobra × Normande, and undefined crossbreds clustered in an intermediate position between the indigenous and European breeds (Figure 3). The history of this crossbred population suggests that these animals are likely F1 crosses but PCA cannot differentiate an F1 from any other cross resulting in approximately 50% indigenous ancestry. Maure and Djakore clustered in an intermediate position between *Bos indicus* and African *B. taurus* ancestral populations, while Gobra, the Gobra × Maure cross, and the Gobra × Guzerat cross spread between these two ancestral populations, showing a wide genetic diversity among individuals.

### Estimation of Heterozygosity

#### Individual Heterozygosity in the Reference Populations

The average individual heterozygosity values for European *B. taurus*, African *B. taurus*, Indian *Bos indicus*, and indigenous reference populations as well as West African crossbred populations are presented in Table 3. Friesian and Jersey cattle populations showed the highest and lowest average heterozygosity of the European dairy breeds with 0.331 (SD = 0.013) and 0.261 (SD = 0.014), respectively. These results are consistent with previous estimates; for example, Mbole-Kariuki et al. (2014) found heterozygosities of 0.33 (SD = 0.01) and 0.25 (SD = 0.03) for Holstein-Friesians and Jersey, respectively.

**TABLE 3:** Average heterozygosities of reference and West African crossbred populations.

Breed	Number of animals	Mean	SD	Minimum	Maximum
Friesian	25	0.331	0.013	0.303	0.360
Guernsey	20	0.268	0.014	0.234	0.291
Holstein	20	0.311	0.015	0.276	0.343
Jersey	20	0.261	0.014	0.227	0.286
Montbéliarde	20	0.295	0.008	0.279	0.303
**Pooled populations**					
European *Bos taurus*	125	0.295	0.030	0.227	0.360
African *Bos taurus*	87	0.198	0.015	0.141	0.218
Bos indicus	105	0.158	0.014	0.110	0.181
Indigenous	105	0.238	0.023	0.129	0.261
Crossbreds*	394	0.328	0.030	0.166	0.370

*Crosses between the local breed Gobra with Holstein-Friesian, Montbéliarde, Normande, and undefined crossbreds.

As expected, the average heterozygosity proportion in crossbred animals was higher (0.3277, SD = 0.030) than in the pooled pure reference and indigenous populations (Table 3). However, the average heterozygosity proportion in crossbreds were lower than in Friesian, which is due to the outlier animal in the crossbred group that showed a low heterozygosity proportion (0.166). The mean heterozygosity of the crossbreds without the outlier is 0.328. This is still somewhat lower than the Friesian heterozygosity, however, the crossbreds have a larger SD (0.029 vs. 0.013) and the median for the crossbreds is slightly higher (0.339) than the mean, indicating somewhat a skewed distribution. Moreover, the maximum heterozygosity of the crossbreds is higher than any of the other populations. The pooled European *B. taurus* and African *B. taurus* populations had an average heterozygosity of 0.295 (SD = 0.030) and 0.198 (SD = 0.015), respectively. *Bos indicus* had a low average heterozygosity of 0.158 (SD = 0.014), which is even lower than in other studies (Kasarapu et al., 2017; Utsunomiya et al., 2019); however, most other studies did not use *Bos indicus* breeds from India but breeds that are known to have a complex breeding history including introgression of *B. taurus* breeds such as Brahman, Nelore, or Gyr. The low heterozygosity level in *Bos indicus* populations is likely due to ascertainment bias of the SNPs on the assay, which seems to be even more pronounced in *Bos indicus* breeds from India. The pooled indigenous animals had an average heterozygosity of 0.238 (SD = 0.023), consistent with the extra heterozygosity expected in admixtures between the African *B. taurus* and *Bos indicus* ancestral populations. Including heterozygosity proportion in the model for genetic evaluation increases the prediction accuracy of traits and it also has the potential to be used in mate selection in order to maximize heterozygosity in the offspring (De Cara et al., 2011; Iversen et al., 2019). A previous study by Mbole-Kariuki et al. (2014) using a medium-density (50k SNPs) dataset reported a lower average heterozygosity level for N’Dama 0.17 (SD = 0.08) than the pooled African *B. taurus*, and a higher average heterozygosity level for Sheko 0.26 (SD = 0.003) compared to the pooled indigenous animals in our study.

#### Ancestral Origin Heterozygosity in West African Crossbreds

**FIGURE 4:**
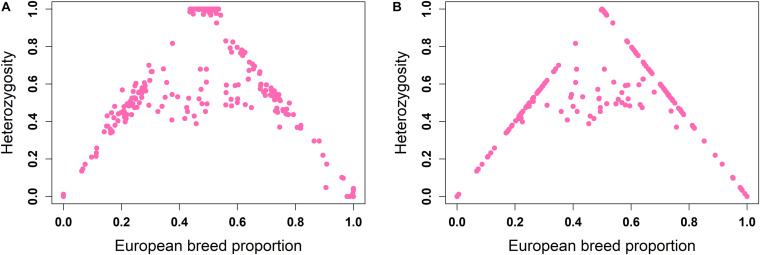
Ancestral origin heterozygosity in West African crossbreds plotted against European breed proportion estimated from a two-way admixture using **(A)** ADMIXTURE and **(B)** LAMP-LD.

**FIGURE 5:**
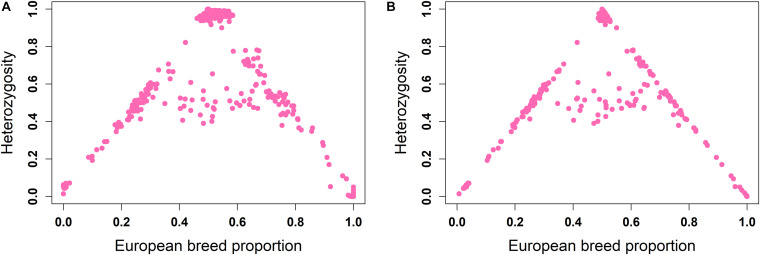
Ancestral origin heterozygosity in West African crossbreds plotted against European breed proportion estimated from a three-way admixture using **(A)** ADMIXTURE and **(B)** LAMP-LD.

The ancestral origin heterozygosity proportions estimated by LAMP-LD are plotted against the estimated European breed proportion from either ADMIXTURE or LAMP-LD for the two-way (Figure 4) and three-way admixture (Figure 5). Animals with low heterozygosity and low (<2% based on the two-way ancestry analysis) European breed proportion are interpreted to be pure indigenous, and animals with low heterozygosity, but high (>98%) European breed proportion are assumed to be pure European dairy breeds. Estimation of European breed proportion using LAMP-LD (Figures 4B, 5B) showed a clearer cluster than the result obtained from ADMIXTURE (Figures 4A, 5A). However, animals that showed up as pure indigenous in all other analyses were estimated by a three-way admixture with LAMP-LD to have a small proportion of European *B. taurus* ancestry. This appears to be due to the model allocating a proportion of the African *B. taurus* ancestry to be European *B. taurus* ancestry.

Theoretically, all crossbreds must sit within the bounds set by the straight lines between F1 animals, with a European breed proportion of 0.5 and ancestral origin heterozygosity of 1.0, and the pure indigenous and European breeds that have European breed proportion of zero and 1.0, respectively, and an ancestral origin heterozygosity of zero. Animals that sit on the outer boundaries are crosses where at least one parent is purebred, whereas animals inside the boundaries result from a mating of two crossbred parents. Based on this assumption, Figure 4B fits the model almost exactly. The plots based on ADMIXTURE estimates of breed proportion (Figures 4A, 5A) fit the model least well because the method of estimating global ancestry by ADMIXTURE differs from that used by LAMP-LD, leading to inconsistencies between the estimate of breed composition (global ancestry).

Although it cannot be seen because of over-position of data points, in Figures 4B, 5B, a high proportion of crossbred animals with almost exactly 50% European breed proportion had ancestral origin heterozygosity of almost exactly 1.0 (Figure 4B) or very close to 1.0 (Figure 5B), which is the heterozygosity expected for F1 crosses. This is visible in Supplementary Figures 1A (two-way admixture) and 1B (three-way admixture), where the number of data points within a particular area of the plot is counted and presented by a color gradient to show how many animals occur at each position on the plot. Comparing Figures 4, 5, and Supplementary Figure 1 shows that the three-way ancestry model leads to more variable estimates of European breed proportion by both ADMIXTURE and LAMP-LD, and more variable estimates of ancestral heterozygosity by LAMP-LD. Most notably, the LAMP-LD estimates of ancestral heterozygosity for the putative F1 animals are all almost exactly equal to the expected value of 1.0 when using the two-way ancestry model, whereas the estimates from the three-way ancestry model, while mostly still close to 1.0, include estimates as low as 0.9.

Supplementary Figure 1 shows that there are clusters of animals on the outer boundaries around 25 and 75% European breed proportions, respectively. These are most likely backcrosses of F1 animals to pure indigenous or pure European animals, which are expected to have European breed proportions that vary around 25 and 75%, and heterozygosities that vary around 0.5 because of a random sampling of gametes from the parents. As most clearly seen in Figure 4B, the majority of animals sit on the boundary lines indicating that in this crossbred population, the majority of animals result from a mating involving at least one purebred parent rather than *inter-se* matings among crossbred animals. This is consistent with the fact that these crossbred dairy populations are relatively recently established and are expanding (K. Marshall, personal communication).

**FIGURE 6:**
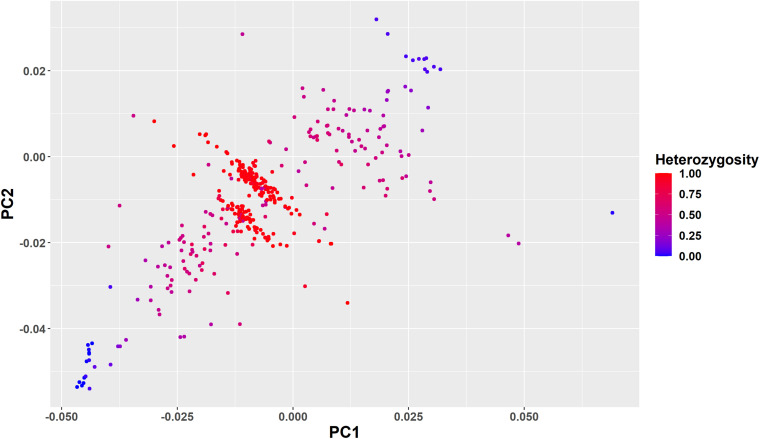
Plots of PC1 vs. PC2 for all West African crossbred animals showing their ancestral origin heterozygosity as color code from red (heterozygosity = 1) to blue (heterozygosity = 0).

To further clarify the genetic structure of the crossbred animals clustered in the intermediate position of the PC plot in Figure 3, we color-coded the individuals based on ancestral origin heterozygosity (Figure 6). This confirms that the majority of animals in the two bands in the middle of the plot are F1 animals with an ancestral origin heterozygosity of 1.0. The majority of Gobra x Holstein-Friesian crosses were clustered in the first band (between PC1 = −0.025 to 0.000 and PC2 = −0.02 to −0.01), while the majority of Gobra × Montbéliarde and Gobra × Normande crosses were clustered in the other band. A substantial number of undefined crossbred animals were clustered in one or the other of the two bands with ancestral origin heterozygosities close to 1.0, showing that they are Gobra x Holstein-Friesian and Gobra x Montbéliarde F1 crosses.

#### Individual Heterozygosity in West African Crossbreds

**FIGURE 7:**
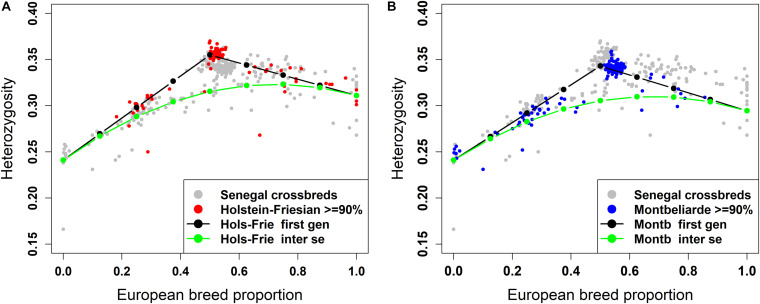
Individual heterozygosity in West African crossbreds plotted against European breed proportion estimated from a three-way admixture using ADMIXTURE. The black and green lines are upper and lower boundaries of expected heterozygosity for **(A)** Holstein-Friesian and **(B)** Montbéliarde crosses.

**FIGURE 8:**
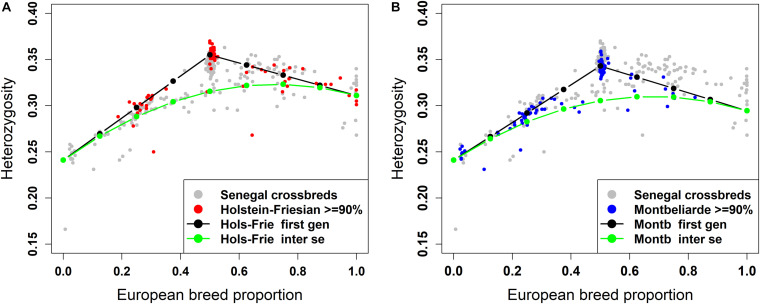
Individual heterozygosity in West African crossbreds plotted against European breed proportion estimated from a three-way admixture using LAMP-LD. The black and green lines are upper and lower boundaries of expected heterozygosity for **(A)** Holstein-Friesian and **(B)** Montbéliarde crosses.

The plot of individual heterozygosity against European breed proportion for the West African crossbred cattle obtained from ADMIXTURE and LAMP-LD using the three-way admixture is shown in Figures 7A, 8A, respectively. For completeness, Supplementary Figure 2 shows the individual heterozygosity against European breed proportion obtained from ADMIXTURE and LAMP-LD using two-way admixture. To avoid duplication, only the results of the three-way admixture are discussed here. The animals in red color in the Figures 7A, 8A have ≥90% of their European breed proportion being Holstein-Friesian, while the animals shown in blue color in Figures 7B, 8B are those having ≥90% of their European breed proportion being Montb liarde.

Across all animals, the individual heterozygosity ranged from 0.166 to 0.37, and the European breed proportion ranged from 0 to 1. There are evident clusters of animals that have high heterozygosity proportions (>32%) and are close to 50% European breed proportion. Virtually all of these animals are those shown to be F1 crosses in the ancestral origin heterozygosity results.

The black lines are the expected heterozygosity proportions for the progeny of crosses involving an average Holstein-Friesian parent (Figures 7A, 8A) or an average Montbéliarde parent (Figures 7B, 8B). The green lines are the expected heterozygosity proportions for the progeny of *inter-se* matings between crossbred animals of identical breed composition. The black lines form a theoretical upper boundary of heterozygosity of crossbred animals, while the green lines are the theoretical lower boundary. Holstein, Friesian and Montbeliarde reference samples were used to obtain the average heterozygosity proportion of the pure Holstein-Friesian and Montbéliarde parental populations, respectively, and then used in obtaining the upper and lower boundaries of the expected heterozygosity. The average heterozygosity of indigenous animals was obtained as the average heterozygosity of animals with <2% European breed proportion based on a two-way ancestry analysis. The average heterozygosity of F1 Holstein-Friesian versus F1 Montbéliarde crossbreds was obtained by identifying F1 animals from the ancestral origin heterozygosity analyses, and matching these to animals whose European breed proportion was ≥90% Holstein Friesian or ≥90% Montbéliarde.

The average individual heterozygosity proportions for the parental indigenous, Holstein-Friesian, and Montbéliarde populations were 0.241 (ranged from 0.166 to 0.258), 0.311 (ranged from 0.276 to 0.343), and 0.295 (ranged from 0.279 to 0.303), respectively. The mean and range of indigenous animals include a single outlier with very low heterozygosity, which the PC plots and admixture analyses indicated was a pure *Bos indicus* animal; most likely one of the pure Guzerat animals known to have been imported into the sample area from Brazil. This outlier was assigned as crossbred in our data using farmers’ assessment of breed composition based on the external appearance of the animal, however, our genomic breed composition prediction methods showed the opposite. Previously, Weerasinghe (2014) tested the extent of farmers knowledge on the ability to identify the breed composition of the East African crossbreds and concluded that farmers have a poor understanding of the breed composition of their animals.

The Holstein-Friesian crosses showed a higher average heterozygosity proportion than the Montbéliarde crosses, and this leads to higher upper and lower boundaries of heterozygosity of Holstein-Friesian crossbreds. The fit to the data is clearly better in Figure 8 than Figure 7, due largely to LAMP-LD providing more accurate estimates of European breed proportion than ADMIXTURE. However, the fit to the data, in general, is very poor in both figures, with a high proportion of animals sitting outside the upper and lower boundaries of heterozygosity. This is due primarily to the large variation in heterozygosity among purebred ancestors. This variation can be expected among ancestors in any crossbred population. Thus, in marked contras to ancestral origin heterozygosity, individual heterozygosity will provide a very poor measure of heterozygosity caused by crossbreeding and hence very poor estimates of heterosis of performance when used in analyses of additive and heterosis effects in this, and by extrapolation other crossbred populations. An additional factor in the current population is the small proportion of crosses resulting from pure Guzerat or Guzerat x indigenous ancestors. These can be seen in Figures 7, 8 as animals appearing well below the green line. They are also evident in Figure 2 as animals with zero or well below expected African *B. taurus* ancestry, and in the PC plot (Figure 3) as animals well below the distribution of points for most crossbreds. A few animals that are scattered well below the expected lower boundaries, such as an animal with European breed proportion around 65% and Holstein-Friesian proportion ≥90%, might be a cross among close relatives resulting in high inbreeding.

Overall the results on ancestral origin versus individual heterozygosity show the clear superiority of ancestral mapping heterozygosity to infer ancestry of individual animals and as an estimate that can be used to obtain estimates of additive and heterosis effects in crossbred populations. The ancestral haplotype inference from LAMP-LD also produced estimates of European breed proportion that were more consistent with expectations than ADMIXTURE, which showed an upwards bias of estimates of European breed proportion for animals with very low European breed proportions when using a three-way analysis. Although not tested here, it is possible that this bias in estimates of European breed proportion could be corrected by rescaling the Admixture estimates. But deriving the rescaling method would require that either the true ancestral bred proportions were known, which will never be the case, or that better estimates are available such as those obtained from LAMP-LD. So, in most cases it seems preferable to simply use the LAMP-LD estimates directly.

### Accuracy of Genotype Imputation in West African Cattle Populations

#### Genotype Imputation From Low-Density to Medium-Density

**FIGURE 9:**
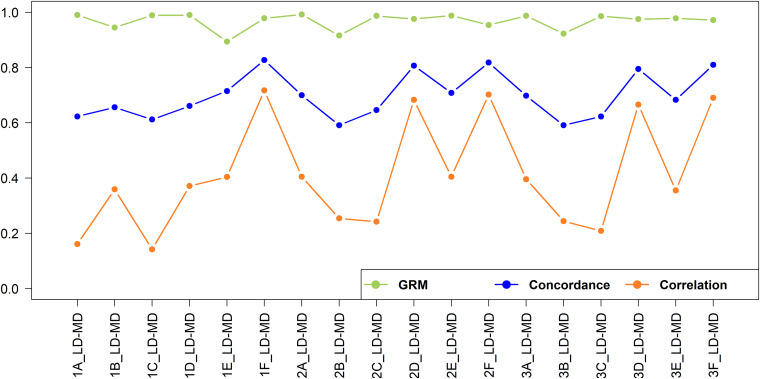
Genotype imputation accuracy and GRM correlation between real and imputed genotypes for imputation from low to medium-density.

The concordance and correlation of imputation from low to medium density under various scenarios are shown in Figure 9. As expected, for all scenarios the concordance was higher and much less varying than the correlation. Several authors report both the correlation and concordance rate to compare the accuracy of imputation in cattle populations (Dassonneville et al., 2012; Berry et al., 2014; Aliloo et al., 2018). However, using the concordance rate as the best measure of imputation accuracy may be misleading because it was found to inflate accuracy for rare and low-frequency variants due to chance concordance or chance agreement (Hickey et al., 2012). To illustrate the effects of MAF on imputation accuracy, the value of correlation and concordance of imputed SNPs for the 2F_LD-MD scenario were plotted against the MAF (Supplementary Figure 3). A higher concordance value was achieved for SNPs with low MAF and the value declined as MAF increased, while the correlation value was not influenced by MAF. This is due to a high chance of correctly assigning rare alleles based on the allele frequencies of the population by transferring the major allele as the missing allele.

In this study, the concordance was included for comparison with published literature but is not further discussed here as it represents a poor measure of the accuracy of imputation. From this point onwards, the estimate of the accuracy of imputation is the correlation of imputed versus true SNP genotypes. A total of 23,606 SNPs were imputed from low to high-density dataset. The imputation accuracies from low to medium-density were very low for all scenarios, ranging from 0.142 to 0.717. The best-case scenario (1F_LD-MD; *r* = 0.717), has an *r*^2^ of only 0.514; i.e., only 51.4% of the variation in SNP genotypes is accounted for imputation. Low accuracy of imputation may not be a major problem for some applications. Figure 9 also shows the correlation of the off-diagonal elements to the GRM built with imputed versus true genotypes, and these range from 0.894 to 0.992. This suggests that genomic estimated breeding values (GEBV) resulting from imputed versus real genotypes should be very highly correlated in many cases (Wu et al., 2016; Aliloo et al., 2018).

The imputation accuracies for the crossbreds were relatively higher when crossbreds or a combination of indigenous and crossbred populations were used as the reference sets. For Scenario 1, where East African populations were used as a reference, the accuracy was higher within the East African crossbred populations (Scenario 1F_LD-MD), while it was very low for imputation of West African indigenous populations (Scenario 1A_LD-MD and Scenario 1C_LD-MD). The accuracy improved when the imputation was performed within the West African indigenous (Scenario 2A_LD-MD) and crossbreds (Scenario 2D_LD-MD). The inclusion of East African indigenous to West African indigenous reference set did not improve the imputation accuracy (Scenario 2A_LD-MD versus Scenario 3A_LD-MD), while adding East African crossbreds to the West African crossbred reference set (Scenario 3D_LD-MD) resulted in a slight decrease in imputation accuracy.

Imputation accuracies are generally expected to be reasonably high for European dairy breeds, given that the SNP assays were in part designed for use in European *B. taurus* breeds and that training of imputation is often based on large sample sizes. Several authors reported an imputation accuracy (correlation) greater than 0.9 in European dairy breeds (Dassonneville et al., 2012; Mulder et al., 2012; Berry et al., 2014). This allows their widespread use for imputation and then the application to generate genomic EBVs, allowing lower cost and wider application of genotyping in genetic improvement. The accuracy in our African crossbred populations never approaches that found in European dairy breeds, even where the reference data involves many thousand animals sampled from the same population, as in the use of East African data to impute East African crossbred genotypes. We, therefore, infer that a new assay will need to be designed if low-density assays are to be reliable for use in genetic analyses of African crossbred dairy cattle. Although we have much less data on indigenous breeds than crossbreds, and hence cannot clearly differentiate the impact of low sample size versus poor assay design, it is reasonable to infer that newly designed assays will also be required for use in African indigenous breeds. Another reason could be the higher genetic diversity in African indigenous breeds compared to European dairy breeds (Gebrehiwot, 2020; Gebrehiwot et al., 2020), which might complicate imputation and reduces accuracy.

#### Genotype Imputation From Medium-Density to High-Density

**FIGURE 10:**
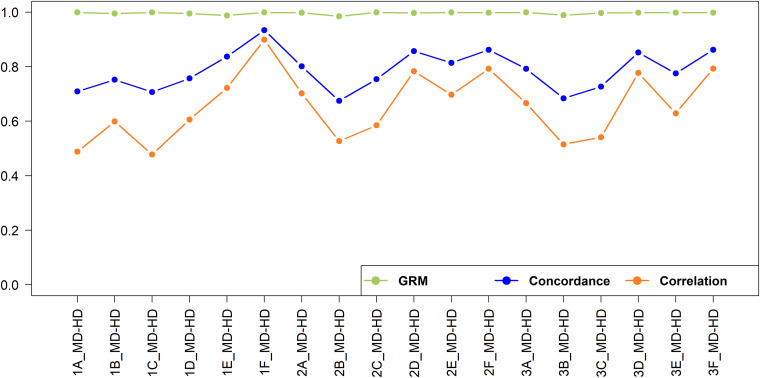
Genotype imputation accuracy and GRM correlation between real and imputed genotypes for imputation from medium to high-density.

The imputation concordance and correlation and the correlation of off-diagonal elements of the GRM for imputation from medium to high density are shown in Figure 10. A total of 592,660 SNPs were imputed from medium to high-density dataset. As expected given the substantially larger number of SNPs involved and hence smaller distance between adjacent SNPs, the imputation accuracy was always higher than when imputing from low to medium density.

In general, the accuracy was higher when imputation was performed within the geographical region than across geographical regions. This observation was also made in European dairy breeds, were a Holstein reference population yielded a lower imputation accuracy in German Black Pied cattle, despite providing a larger reference, compared to using a reference population of the same breed (Korkuæ et al., 2019). The accuracy was the highest (correlation = 0.899) when East African crossbreds were used as a reference set to impute East African crossbreds (Scenario 1F_MD_HD). This was because of the larger size of the reference set, and the reference set being sampled from the same population as the target set. Recently, Aliloo et al. (2018) reported a slightly higher imputation accuracy (correlation = 0.927) using a combined data of East African crossbred cows and bulls compared to the accuracy obtained in our study, which the data used here is a subset of the populations used by these authors. The slightly higher accuracy is likely due to the higher number of SNPs in their medium-density (dataset (42k SNPs) compared to the number available in our study (29k SNPs).

In West African populations, the accuracy of imputation was higher for crossbreds than the indigenous populations. This is in concordance with (Rowan et al., 2019) who reported that a multi-breed composite reference significantly increased imputation accuracy compared to a within-breed reference population. The highest correlation (0.702) for West African indigenous animals was found when a West African indigenous reference population was used (Scenario 2A_MD-HD), while the lowest correlation (0.478) was found when an East African indigenous reference population was used (Scenario 1C_MD-HD). The lower imputation accuracy for the indigenous populations compared to that of crossbreds is likely due to a combination of the smaller reference population size and the relatively high effective population sizes (*Ne*) and high genetic diversity in the African indigenous breeds (Gebrehiwot, 2020; Gebrehiwot et al., 2020). The accuracy of imputation for the indigenous populations would likely have been improved if the imputation had been performed within indigenous breeds, hence maximizing the shared LD between SNPs, rather than pooling all the indigenous data together; but sample sizes were too small here to test that hypothesis. The research here does not directly identify a target number to be genotyped but by extrapolation from imputation in the East African crossbred populations (Aliloo et al., 2018) at least 1,000 animals will be needed.

The addition of East African indigenous data to the West African indigenous reference data (Scenario 3A_MD-HD) and the addition of the East African crossbred data to the West African crossbred reference data (Scenario 3D_MD-HD) decreased the accuracies of prediction of West African indigenous and West African crossbreds by 4 and 1%, respectively. Brøndum et al. (2012) reported a similar reduction of imputation accuracy in a Holstein population when Danish, Swedish and Finnish Red cattle populations were added to the Holstein-Friesian reference set. This is likely due to a lack of consistent LD phase between these populations. In all scenarios, adding indigenous and crossbred reference data to impute crossbreds or adding crossbred data to indigenous data to impute in indigenous animals either decreased accuracies or increased only slightly (<3%) compared to use of crossbred or indigenous reference data alone. These small changes in accuracy, even when a large amount of data was added (e.g., Scenario 1C_MD-HD and 1D_MD-HD versus Scenario 1A_MD-HD and 1B_MD-HD, respectively), indicate that the additional data had little or no shared LD phase with the target population. Taken together, the results show that in order to obtain reasonably high accuracy of imputation within African indigenous or crossbred populations substantial reference data will need to be collected for the target populations because reference data from indigenous or crossbred populations from other regions of Africa generally provide poor accuracy of imputation.

The correlations of the off-diagonal elements of the GRMs constructed using real versus imputed genotypes (Figure 10) were all above 0.985. This is consistent with previous findings that even with a high error rate in genotype imputation, the genomic prediction accuracy still can be high (Wu et al., 2016; Aliloo et al., 2018). Our study further assessed the correlations of off-diagonal elements of the GRMs constructed using the real low-density versus medium-density and medium-density versus high-density genotypes for East African indigenous and crossbred populations and obtained correlation of 0.958 and 0.990 and 0.938 and 0.987, respectively. The high correlations among off-diagonal elements of GRMs from different density panels implies that the loss in genetic gain to implement genomic prediction using low or medium-density datasets compared to high-density genotypes is small in the East African cattle populations. Previously, Habier et al. (2009) and Cleveland et al. (2010) supported the feasibility of undertaking genomic prediction based on low-density genotypes for practical implementation, and the cost-efficiency of low-density genotypes allows a much larger proportion of the population to be included in the genomic evaluation procedure (Wiggans et al., 2012).

Overall, genomic information from high-density genotypes provides the opportunity to increase the rate of genetic progress in breeding programs (Hayes and Goddard, 2001). Though the price of high-density marker arrays is continually reducing, genotyping cost still is one of the main limiting factors for cost-efficient genomic applications. This high cost could be an issue in developing countries in Africa, where financial resources are very limited for the key stakeholders, such as smallholder dairy farmers. Therefore, a strategy that is used to overcome the cost limitations is to genotype a sufficiently large number of reference individuals from a given population with higher density or fully sequenced while the majority will be genotyped with lower density. This cost-effective strategy provides reliabilities of GEBVs that are similar to those obtained if selection candidates were genotyped with the higher-density chip (Khatkar et al., 2012; Mulder et al., 2012).

## Conclusion

This study shows that ancestral heterozygosity can be estimated with high accuracy in African crossbred populations and will be far superior to the use of observed individual heterozygosity for estimating heterosis in such crossbred populations. The population-based imputation results highlighted the effects of different reference populations, SNP density, and sample size on imputation accuracy. It has been hoped by research groups working in Africa that high imputation accuracy might be achieved in African populations by using large-scale imputation information from other populations to impute in populations in which there is limited high-density genotype information, as has often been found to be possible for different breeds in developed countries. Unfortunately, the results show clearly that it was not possible to achieve high imputation accuracy in West African crossbred or indigenous populations based on large reference data sets from East Africa, and so larger population-specific genotype samples, especially considering the larger genetic diversity of African indigenous cattle, will be required to achieve high accuracy. This study provides a strong foundation to integrate genotype imputation into routine genomic evaluation pipelines for African cattle populations as a cost-effective way to boost the power of genomic-based genetic improvement.

## Data Availability Statement

Data were sourced from the public domain and privately held databases as detailed in the manuscript. In most cases, the data held privately is available on request to the institution owning the data.

## Ethics Statement

Ethical review and approval was not required for the animal study because Not applicable. Data sourced from previous studies.

## Author Contributions

NG, JG, and HA conceived and designed the outline for the study. KM supervised the original Senegal study and arranged for the collection and genotyping of new Senegal samples used here. AM collected the 141 crossbred samples. NG performed the data analysis and drafted the manuscript. HA and MA gave methodological support. NG, JG, ES, HA, and KM interpreted the results. All authors read and approved the final manuscript.

## Conflict of Interest

The authors declare that the research was conducted in the absence of any commercial or financial relationships that could be construed as a potential conflict of interest.

## Supplementary Material

Supplementary Figure S1 Ancestral origin heterozygosity against European breed proportion of crossbred animals using LAMP-LD based on **(A)** two-way admixture and **(B)** three-way admixture, showing the number of animals clustered at a particular location. Note that the clustering algorithm causes exaggerated shifts of the centroid of each point on the plot compared to Figures 4B, 5B.

Supplementary Figure S2 Individual heterozygosity in West African crossbred cattle plotted against European breed proportion estimated using two-way admixture using **(A)** ADMIXTURE and **(B)** LAMP-LD.

Supplementary Figure S3 The value of correlation and concordance of imputed SNPs against the MAF for the 2F_LD-MD scenario.
