# Supplementary figures and images for: Inference of Ancestries and Heterozygosity Proportion and Genotype Imputation in West African Cattle Populations
Source: Front Genet. 2021 Mar 23;12:584355. doi: 10.3389/fgene.2021.584355 (PMC8025404; doi:10.3389/fgene.2021.584355)

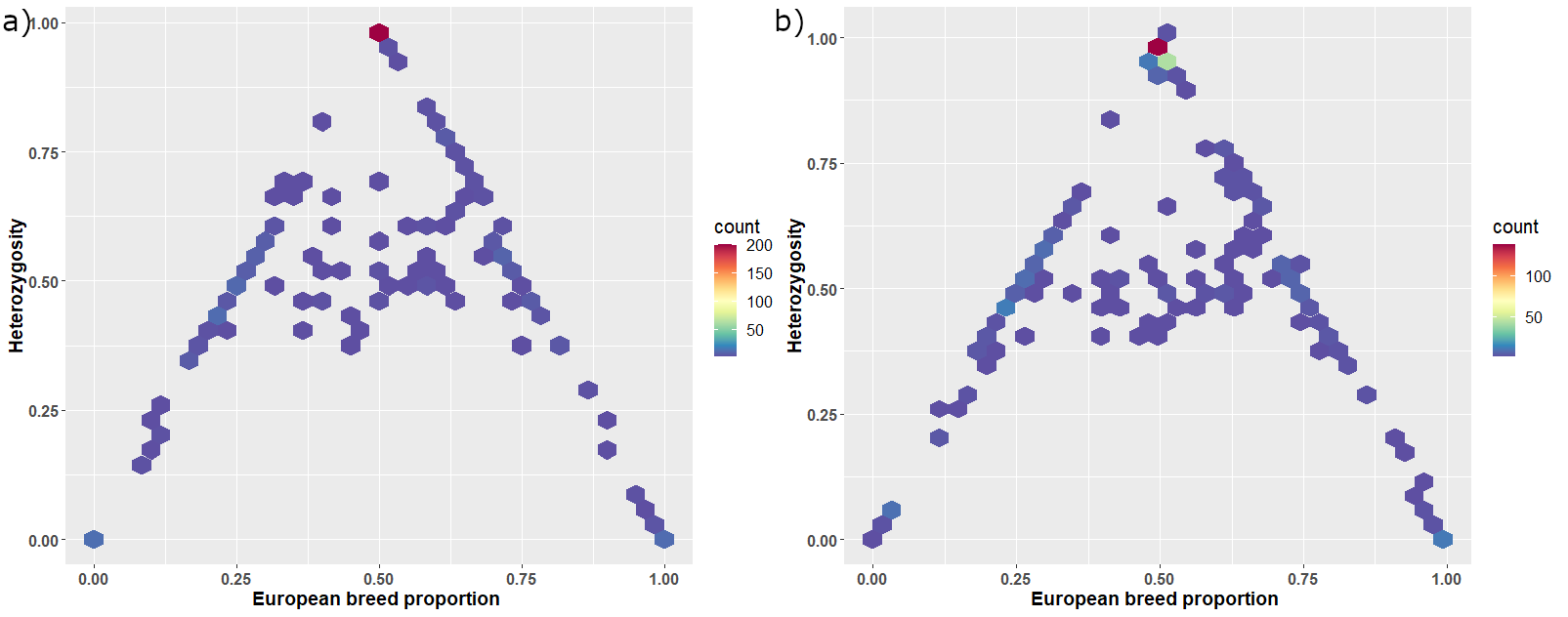

Supplement: Supplementary Figure S1 — Ancestral origin heterozygosity against European breed proportion of crossbred animals using LAMP-LD based on (A) two-way admixture and (B) three-way admixture, showing the number of animals clustered at a particular location. Note that the clustering algorithm causes exaggerated shifts of the centroid of each point on the plot compared to Figures 4B, 5B. [file Image_1.TIFF]

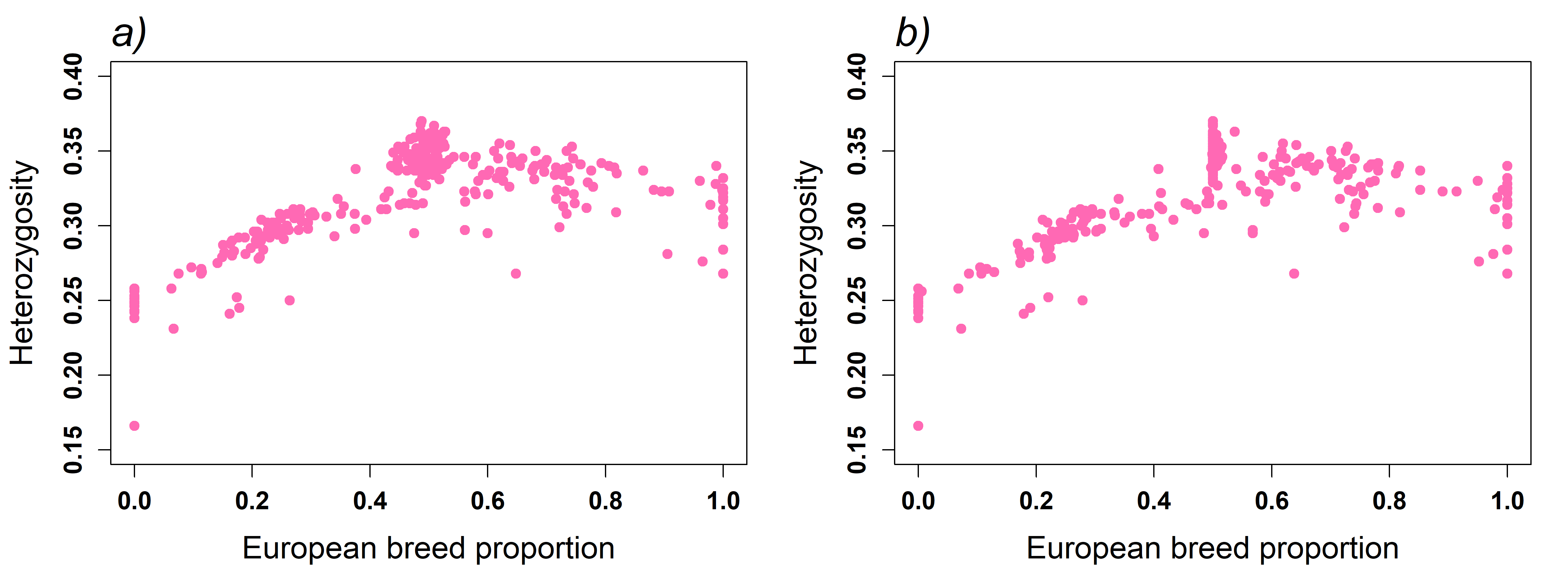

Supplement: Supplementary Figure S2 — Individual heterozygosity in West African crossbred cattle plotted against European breed proportion estimated using two-way admixture using (A) ADMIXTURE and (B) LAMP-LD. [file Image_2.TIFF]

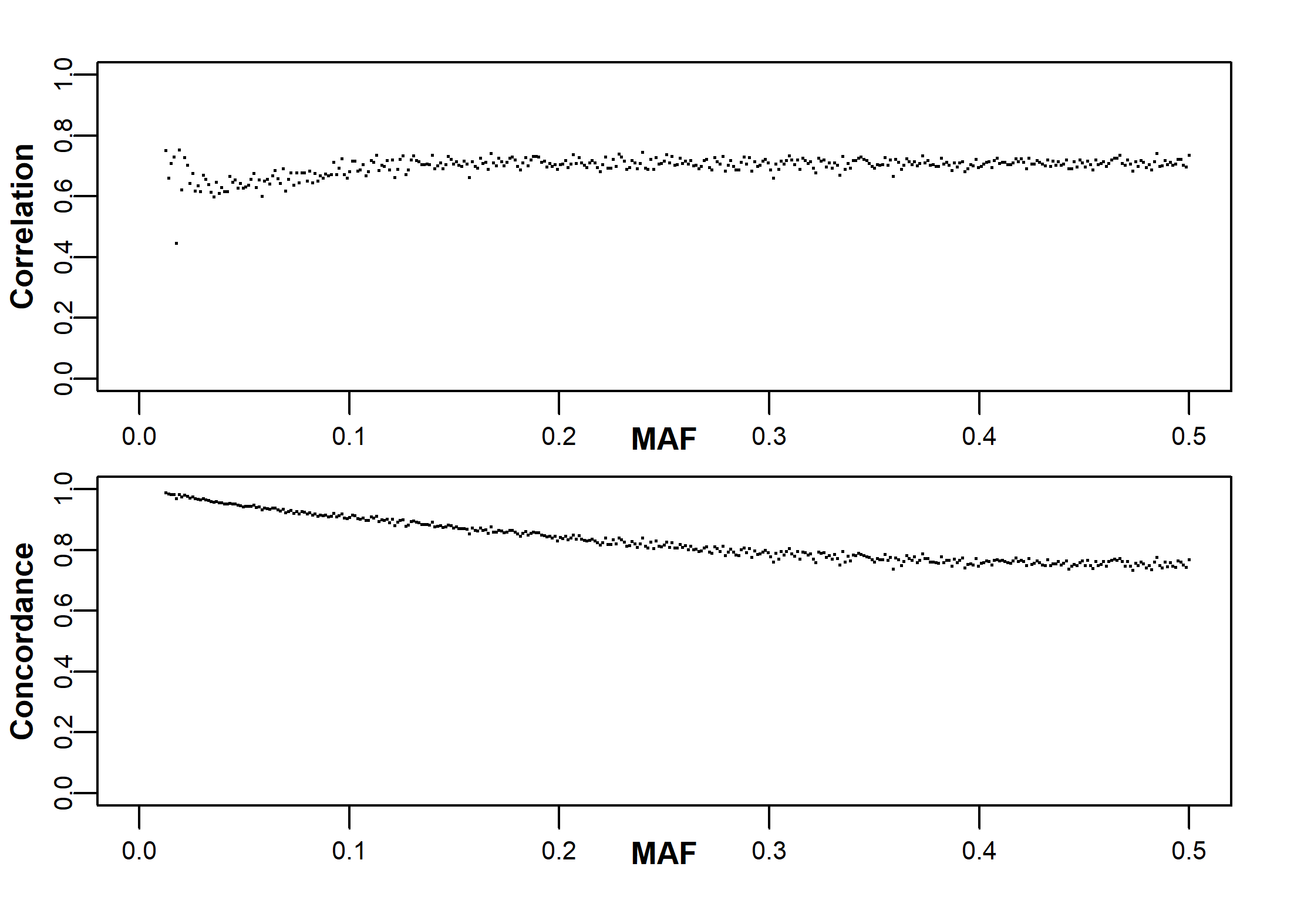

Supplement: Supplementary Figure S3 — The value of correlation and concordance of imputed SNPs against the MAF for the 2F_LD-MD scenario. [file Image_3.TIFF]
